# An inconvenient dataset: bias and inappropriate inference with the multilevel model

**DOI:** 10.1007/s11135-013-9865-x

**Published:** 2013-06-06

**Authors:** Samuel R. Lucas

**Affiliations:** Department of Sociology, University of California, Berkeley, Berkeley, CA USA

**Keywords:** Multilevel modeling, Sample design, Non-probability sample, Probability sample, FMP sample

## Abstract

The multilevel model has become a staple of social research. I textually and formally explicate sample design features that, I contend, are required for unbiased estimation of macro-level multilevel model parameters and the use of tools for statistical inference, such as standard errors. After detailing the limited and conflicting guidance on sample design in the multilevel model didactic literature, illustrative nationally-representative datasets and published examples that violate the posited requirements are identified. Because the didactic literature is either silent on sample design requirements or in disagreement with the constraints posited here, two Monte Carlo simulations are conducted to clarify the issues. The results indicate that bias follows use of samples that fail to satisfy the requirements outlined; notably, the bias is poorly-behaved, such that estimates provide neither upper nor lower bounds for the population parameter. Further, hypothesis tests are unjustified. Thus, published multilevel model analyses using many workhorse datasets, including NELS, AdHealth, NLSY, GSS, PSID, and SIPP, often unwittingly convey substantive results and theoretical conclusions that lack foundation. Future research using the multilevel model should be limited to cases that satisfy the sample requirements described.

## Introduction

The multilevel model (MLM) has become a staple of social research. Researchers have used it to explore and explain cross-national fertility differences (Mason et al. 1983), effects of track location on student achievement (Gamoran 1992), life circumstance effects on criminal conduct (Horney et al. 1995), labor market restructuring and gender (McCall 2000), grandparents’ effects on child mortality (Beise and Voland 2002), childcare availability effects on fertility decisions (Hank and Kreyenfeld 2003), schools’ varying racial differences in college preparatory course-taking (Lucas and Berends 2007), the power of community characteristics on community attachment (Flaherty and Brown 2010), effects of race and sex discrimination on earnings (Lucas 2013b), and much more.^1^ In these and other MLM analyses researchers have estimated statistical models more consistent with their multilevel theories, facilitated more effective partition of variance into multiple levels, and improved the accuracy of standard errors. These features make the MLM a powerful research tool.

However, every tool has attendant requirements. Eager to assess a vast set of multilevel substantive and theoretical issues, analysts have applied the model broadly. Yet, I contend, some datasets on which the MLM has been estimated are non-probability samples for the MLM. If so, estimators are biased and the tools of inferential statistics (e.g., standard errors) are inapplicable, dissolving the foundation for findings from such studies. Further, this circumstance may not be rare; the processes transforming probability samples into problematic samples for the MLM may be inconspicuous but widespread. If this reasoning is correct, many published MLM study findings are likely wrong and, in any case, cannot be evaluated, meaning that, to the extent our knowledge depends on that research, our knowledge is compromised.

The general elusiveness of probability samples and that complex models can escalate sample demands are well-known facts. With respect to the latter, the advent of event history analysis required analysts to appreciate that cross-sectional data produces erroneous estimates for life-cycle events (Morgan and Liao 1985; Freedman et al. 1988). Analysts further established that combining cross-sections can easily exacerbate these biases (e.g., Allison 1982; Lucas et al. 2011). As another example, Clogg and Eliason (1987) show that complex sample designs can bias log-linear model estimation. One can neither simply model weighted counts nor ignore sample design; instead, one must make a more complex adjustment.

The conceptual shift the MLM entails is more noteworthy than that implied by complex sampling for log-linear modeling but perhaps less noteworthy than the focus-shift event history analysis necessitated. Study of log-linear, event history, and other models suggests that ignoring models’ sample design demands can lead to bias and inappropriate inference.

With respect to the general elusiveness of probability samples, missing data pose one clear threat (Little and Rubin 2002). Social processes can be equally damaging, transforming probability samples into problematic samples for a given research question. For example, probability samples of adults, such as the U.S. Current Population Survey, provide selected samples for estimating the gender wage gap, because those in the paid labor force are a non-random set of adults. Repairing such samples has been an active area of study for decades (e.g., Heckman 1979; Berk 1983; Stolzenberg and Relles 1990; Breen 1996; Tam 2011) but, even so, solutions are elusive (Winship and Mare 1992).

Missing data and censored, selected, and truncated samples (Breen 1996) have been objects of research because analysts have accepted that these pose serious challenges to inference. Judging from the empirical literature, however, we have yet to clearly establish that probability samples can be *non*-probability samples for the MLM, trace the impact this has on inference, or convey the implications to empirical researchers. This paper, therefore, describes how non-probability samples arise from probability samples in the multilevel context, and, using theoretical argument and Monte Carlo simulations, demonstrates what costs, if any, follow.

To that end, I first relate the MLM, followed by my case for the sample design demands of multilevel modeling, stated first in textual and then in formal terms. Next issues of estimation that might confuse are addressed, followed by review of the didactic MLM literature treatment of sample design. I then convey how several common nationally-representative datasets fail to meet the data demands posited. Afterward, Monte Carlo simulations illustrate costs of violating posited MLM sample requirements, followed by a concluding section.

## The multilevel model

The MLM goes by many different names in multiple fields, and several specifications have been offered. To fix ideas, a multi-equation specification provides one individual-level ($$i)$$ equation containing an outcome variable (Y) and several determinants (X’s). If a coefficient ($$\beta )$$ for a given X is allowed to vary over $$J$$ macro-level units, then an equation at the macro-level may contain coefficients ($$\gamma $$’s) for macro-level factors (Z’s) that may partially determine $$\beta _{j}$$. So, for example, Eqs. 1–2 describe a two-level slopes-as-outcomes model:

1 $$\begin{aligned} \text{Y}_\mathrm{ij} =\beta _0 +\beta _{1\mathrm{j}} \text{X}_{1\mathrm{ij}} +\beta _2 \text{X}_{2\mathrm{ij}} +\beta _3 \text{X}_{3\mathrm{ij}} +\varepsilon _{\mathrm{ij}} \end{aligned}$$

2a $$\begin{aligned} \beta _0 =\gamma _{00}\end{aligned}$$

2b $$\begin{aligned} \beta _{\mathrm{1j}} =\gamma _{01} +\gamma _{11} \text{Z}_{1\mathrm{j}} +\delta _{1\mathrm{j}}\end{aligned}$$

2c $$\begin{aligned} \beta _2 =\gamma _{02}\end{aligned}$$

2d $$\begin{aligned} \beta _3 =\gamma _{03} \end{aligned}$$

$$\begin{aligned} \varepsilon _{\mathrm{ij}} \sim \text{N}( {0,\sigma ^2});\quad \delta _{\mathrm{kj}} \sim \text{N}( {0,\mathbf{T}});\quad \rho _{\varepsilon \delta} =0 \end{aligned}$$

Equation 1 specifies the level-1 equation, whereas Eqs. 2a–2d specify the macro-level (or level-2) equations. $\varepsilon _{ij}$ and $\delta _{1j}$ are individual- and macro-level errors with variance $\sigma ^{2}$ and variance-covariance matrix $\mathbf{T}$, respectively. The level-1 coefficient for X$_{1}$ varies across macro-level units, while the other level-1 coefficients do not. In Eq. 2b the variation in $\beta _{1j}$ is partially associated with macro-level variable Z$_{1}$.

Similarly, Eqs. 3 and 4 constitute a means-as-outcomes model:

3 $$\begin{aligned} \text{Y}_{\mathrm{ij}} =\beta _{0\mathrm{j}} +\beta _1 \text{X}_{1\mathrm{ij}} +\beta _2 \text{X}_{2\mathrm{ij}} +\beta _3 \text{X}_{3\mathrm{ij}} +\varepsilon _{\mathrm{ij}} \end{aligned}$$

4a $$\begin{aligned} \beta _{0\mathrm{j}} =\lambda _{00} +\lambda _{10} \text{Z}_{1\mathrm{j}} +\delta _{0\mathrm{j}}\end{aligned}$$

4b $$\begin{aligned} \beta _1 =\lambda _{01}\end{aligned}$$

4c $$\begin{aligned} \beta _2 =\lambda _{02}\end{aligned}$$

4d $$\begin{aligned} \beta _3 =\lambda _{03} \end{aligned}$$

$$\begin{aligned} \varepsilon _{\mathrm{ij}} \sim \text{N}( {0,\sigma ^2});\quad \delta _{\mathrm{kj}} \sim \text{N}( {0,\mathbf{T}});\quad \rho _{\varepsilon \delta} =0 \end{aligned}$$

Equations 1–4 imply the MLM partitions the variance across levels and estimates more appropriate standard errors for macro-level coefficients.

Equivalently, one can write the MLM as in Eq. 5, which combines Eqs. 1–2:

5 $$\begin{aligned} \begin{aligned} \text{Y}_{\mathrm{ij}} =\gamma _{00}&+\gamma _{01} \text{X}_{1\mathrm{ij}} +\gamma _{11} \text{X}_{\text{1ij}} \text{Z}_{\text{1j}} +\delta _{1\mathrm{j}} \text{X}_{1\mathrm{ij}} +\gamma _{02} \text{X}_{2\mathrm{ij}} +\gamma _{03} \text{X}_{3\mathrm{ij}} +\varepsilon _{\mathrm{ij}}\\&\qquad \varepsilon _{\mathrm{ij}} \sim \text{N}( {0,\sigma ^2});\quad \delta _{\mathrm{kj}} \sim \text{N}( {0,\mathbf{T}});\quad \rho _{\varepsilon \delta} =0 \end{aligned} \end{aligned}$$

and as in Eq. 6, which combines Eqs. 3–4:

6 $$\begin{aligned} \begin{aligned} \text{Y}_{\mathrm{ij}} =\lambda _{00}&+\lambda _{10} \text{Z}_{1\mathrm{j}} +\delta _{0\mathrm{j}} +\lambda _{01} \text{X}_{1\mathrm{ij}} +\lambda _{02} \text{X}_{2\mathrm{ij}} +\lambda _{03} \text{X}_{3\mathrm{ij}} +\varepsilon _{\mathrm{ij}}\\&\qquad \varepsilon _{\mathrm{ij}} \sim \text{N}( {0,\sigma ^2});\quad \delta _{\mathrm{kj}} \sim \text{N}( {0,\mathbf{T}});\quad \rho _{\varepsilon \delta} =0 \end{aligned} \end{aligned}$$

## Probability sampling theory and its implications: textual explication

The theoretical case for MLM sample design demands requires briefly defining representativeness and its implications. Next, three kinds of sample problems, and then three kinds of representativeness, are identified. Afterwards, I partition parameters into six categories and discuss the implications of failure to meet the posited sample design demands for bias and hypothesis testing for each.

### Representativeness and its implications

I define “representative” samples as follows. A set of $n$ cases are collectively representative of some larger collection of N cases when the process of selecting the $n$ cases is such that an unbiased estimator will unbiasedly estimate population parameters. Though one could refer to a set of cases as “representative for estimation of a specific population parameter, $\varphi$,” for efficiency of expression I refer to “representative” samples and “unbiased” samples as well as their opposites.

Probability sampling is the only sampling method to meet the criterion above. Further, hypothesis testing of estimates is justified by properties of probability samples. Estimates from repeated probability sampling from a population form a normal distribution, and the variance of the distribution has known properties (e.g., the well-known property that in the limit 1.96 standard deviations above the mean carves off the highest 2.5 % of estimates). Although non-probability sample analysts may calculate standard errors and other indices using the same formulas probability sample analysts deploy on probability samples, the results lack the properties those statistics have when calculated from a probability sample. Thus, use of standard errors and other inferential techniques with non-probability sampled data is unjustified. Consequently, non-probability sample estimates cannot be tested, inferences cannot be drawn, and thus such work is of extremely limited value. Therefore, confirming one has a probability sample for the intended analysis is important.

### Three familiar sample problems

The sample problem of most familiarity to many social scientists is selection. In a selected sample a random variable ($$Z)$$ determines whether the dependent variable ($$Y)$$ is observed. However, correlates of $$Y$$ ($$X)$$ are observed even when $$Y$$ is not observed. The Heckman (1979) sample selection model is designed for such situations.

In censored samples the value of $Y$ determines whether the value of $Y$ is observed (e.g., $Y$ is observed if it reaches the minimum score needed for program admission), otherwise all that is known is that the value of $Y$ did not meet the criterion. $X$ is observed for all cases, whether $Y$ is observed or not. The Tobit model (Tobin 1958) is designed for such situations.

In truncated samples, the value of $Y$ is observed only for cases that satisfy some criterion on $Y$. Further, $X$ is observed only for cases in which $Y$ is observed. The truncated regression model (Hausman and Wise 1977) is designed for such cases.

There are models to address each problem above. Even so, repairing damaged samples can be challenging. Alas, as we shall see, one may fail to meet MLM sample demands in additional and complex ways, suggesting repair, if needed, may be even more elusive.

### Types of representativeness

Fixing ideas using the two-level case, we need note three kinds of representativeness.^2^ First, for a given geographic or social level of analysis, I define *context-*un*representative micro-level probability sampling* as producing samples of micro-level units (e.g., U.S. residents) that constitute a probability sample for the larger entity (e.g., the U.S.) but, despite their location in specific contexts, the micro-level units do not represent their contextual peers (e.g., fellow Minnesotans).

In contrast, I define *context-representative micro-level probability sampling* as producing samples in which sampled micro-level units represent their peers in lower level context(s) in which they were sampled. Because there are multiple potential geographic and social levels of analysis, a sample may have context-representative probability sampling for some geo-social levels of analysis while having context-unrepresentative sampling for others. For example, in a city-size stratified national probability sample, MLM analysts using cities as a macro-level would be fine, but those using states as the macro-level might be using Detroit residents to represent the entire state of Michigan, producing erroneous results. Which geo-social contexts have context-representative samples depends on sample design. Although this sampling theory observation seems clear, below I show that some published MLM analyses violate it without comment.

Third, I define *macro-level probability sampling* as occurring when the macro-level units in the sample represent a population of macro-level units (e.g., states sampled representing all states, schools sampled representing all schools).

A sample satisfying the latter two criteria is a *fully multilevel probability (FMP) sample*.

A simple random sample (SRS)—a sample where all target population members have equal and independent chances of selection—is a FMP sample for units and geo-social contexts appropriate for the target population. For example, an SRS of 17-year-old U.S. *students* is a FMP sample for same-aged students in schools, states, districts, and other aggregations of students (e.g., school catchment areas), but not for same-aged children in neighborhoods, because, for example, some neighborhood children do not attend school. If the target population fully covers a conceptual population, then for that conceptual population SRS’s are FMP samples for appropriate geo-social levels of analysis. Alas, most large-scale survey data collection uses complex probability sampling that does not satisfy SRS criteria.^3^ When complex sampling is used, determining whether a sample is a FMP sample for a given geo-social level hinges on details of the complex sample design.

Certainly, complex sample designs offer advantages.^4^ Yet, they can make respondents unrepresentative for contextual dimensions incidental to the sample design. For example, if cities are stratified by size and sampled, sampled persons may represent peers in cities in their stratum rather than peers in their state. If so, sampled Cleveland residents would represent Chicago residents more than they represent residents of, for example, Shaker Heights, Ohio, a Cleveland suburb. Thus, complex sample designs entail a “who represents whom” trade-off.

### Types of MLM parameters

The MLM produces many types of parameters, which can be classified in many different ways. We allocate coefficients of Eqs. 1–4 to 6 different classes (see Table 1).

**Table 1:** Six primary types of parameters estimated in common multilevel models

Class	Name	Example
Level-2 parameters		
A	Macro slopes of slopes	$\gamma _{11}$
B	Macro slopes of intercepts	$\lambda _{10}$
C	Macro-adjusted Micro-level slopes	$\gamma _{01}$
D	Macro-adjusted intercepts	$\lambda _{00}$
Level-1 parameters		
E	Micro-level intercepts	$\beta _{00}=\gamma _{00}$
F	Micro-level slopes	$\gamma _{02},\,\gamma _{03,}\lambda _{01,}\lambda _{02,}$ and $\lambda _{03}$

*Bias and inference for class A coefficients*. One may conceptualize the class A coefficient, $\gamma _{11}$, as a population regression coefficient for an equation with $\beta _{1j}$ as the outcome. Given probability sampling theory, to draw inferences concerning class A coefficients should require macro-level probability sampling.

Further, the dependent variable, $\beta _{1j}$, depends at least partly on the level-1 units within the respective macro-level unit. In general, in order for $\text{E}(\hat{\beta}_{1\mathrm{j}} )=\beta _{1\mathrm{j}}$ the sampled micro-level units within unit $j$ must represent all micro-level units in unit $j$. This condition necessitates probability samples within each macro-level unit $j$, i.e., it requires context-representative micro-level probability sampling. Thus, the class A MLM coefficient should require probability samples (or censuses) of micro-level units within each macro-level unit *and* probability samples (or censuses) of macro-level units, i.e., it should require fully multilevel probability samples (or censuses).

*Bias and inference for class B and class C coefficients*. Class B coefficients reflect the direct impact of macro-level factors on micro-level outcomes, while class C coefficients are the micro-level slopes after controlling for the macro-level factors relevant for the slope. Designs that hamper class A coefficients should negatively affect these coefficients, because class B and class C coefficients are as heavily dependent on the grouping design as are class A coefficients.

*Bias and inference for class D coefficients*. Class D coefficients are intercepts adjusted on the basis of macro-level factors. Certain parameterizations can dissolve the means-as-outcomes/slopes-as-outcomes distinction, thereby dissolving the class C/class D distinction. Thus, we should expect that whatever causes problems for class C coefficients also harms class D coefficients.

*Bias and inference for class E and class F coefficients*. What distinguishes Class E and F coefficients in Eqs. 1–4 is that they are estimated with no reference to the structure of nesting, and do not vary across contexts. Thus, macro-level variables cannot explain variation in these non-varying parameters. Accordingly, class E and F coefficients should be unbiased and standard errors should be correct, regardless of whether the sample contains probability samples for smaller contexts or a probability sample of macro-level entities.^5^

## Theorized data requirements for multilevel estimation: formal explication

The above observations can be given a more formal basis. Using the slopes-as-outcomes model, I treat context-representative micro-level probability sampling first, followed by macro-level probability sampling. Results apply to the MLM generally. The analysis treats population parameters, not estimates, on the reasoning that if analysts seek to access one population parameter, but actually access another, estimation of the correct population parameter is compromised before estimation algorithms enter the picture. And, if an incorrect population parameter is accessed, precision estimates for the estimated parameter are irrelevant for the question the analyst sought to ask. Thus, if we establish that MLMs on non-FMP samples lead analysts to access a parameter other than the one they seek to access, we simultaneously establish that the standard error is inappropriate and thus inference for non-FMP samples is indefensible.

### Context-representative micro-level probability sampling

Consider a probability sample designed to represent some large entity (e.g., a nation). But sampled units (henceforth people/persons) are lodged in contexts. Owing to the sample design each context $$j$$ is composed of two groups of persons–sampling-reachable ($$r)$$ and sampling-unreachable ($$u)$$. The proportion of $$r$$ (p) and $$u$$ (1 $$-$$ p) varies across contexts. Further, persons’ allocation to group $$r$$ or $$u$$ is not random, the determinants of assignment may be unknown, and allocation processes may vary across contexts. Thus, groups $$r$$ and $$u$$ differ in unknown yet systematic ways, such that group $$r$$ provides no information on the parameters for group $$u$$.

Even so, given Eqs. 1–2 fixed micro-level population parameters (classes E and F) are estimated unbiasedly and their standard errors apply. However, true context-specific population parameters are actually mixtures as in the following:

7 $$\begin{aligned} \beta _{1\mathrm{j}} =\text{p}_\mathrm{j} \beta _{\mathrm{1j},\mathrm{r}} +( {1-\text{p}_\mathrm{j}})\beta _{\mathrm{1j},\mathrm{u}} \end{aligned}$$

If one proceeds with the MLM one essentially treats $\beta _{1\mathrm{j,r}}$ as if it is $\beta _{1\mathrm{j}}$. Expressed as a function of the true population parameter, in reality:

8 $$\begin{aligned} \beta _{\mathrm{1j,r}} =( {\text{p}_\mathrm{j} \beta _{\mathrm{1j},\mathrm{u}} -\beta _{\mathrm{1j,u}} +\beta _{\mathrm{1j}}})/\text{p}_\mathrm{j} \end{aligned}$$

which is not in general equal to $\beta _{1\mathrm{j}}$. Using $\beta _{1\mathrm{j,r}}$ as if it is $\beta _\mathrm{1j}$ is mistaken, for:

9 $$\begin{aligned} \beta _{\mathrm{1j}} -\beta _{\mathrm{1j, r}} =\text{p}_\mathrm{j} \beta _{\mathrm{1j, r}} +\beta _{\mathrm{1j,u}} -\text{p}_\mathrm{j} \beta _{\mathrm{1j, u}} -\beta _{\mathrm{1j,r}} \end{aligned}$$

which is not in general zero. Equation 9 indicates it will be difficult to establish the magnitude and sign of the difference between $\beta _\mathrm{1j,r}$ and $\beta _\mathrm{1j}$. First, to identify magnitude and sign one needs information about the unreachable sub-population in each context. By definition, one has no information on that sub-population. Second, the unknown bias varies by context as a function of p$_\mathrm{j},\,\beta _\mathrm{1j,r}$, and $\beta _\mathrm{1j,u}$, and its unknown overall value may be misleading, for large context-specific biases may exist when overall bias is low.

Use of $\beta _\mathrm{1j,r}$ for $\beta _\mathrm{1j}$ causes further problems, for Eq. 2b becomes:

10 $$\begin{aligned} \beta _{\mathrm{1j,r}} =( {\text{p}_\mathrm{j} \beta _{\mathrm{1j,u}} -\beta _{\mathrm{1j,u}} +\beta _{\mathrm{1j}}})\,/\,\text{p}_\mathrm{j} =\gamma ^*_{01} +\gamma ^*_{11} \text{Z}_{\mathrm{1j}}+\delta ^*_{\mathrm{1j}} . \end{aligned}$$

For Eq. 10 to produce the sought level-2 population parameters:

11a $$\begin{aligned} \gamma ^*_{01} =\gamma _{01}\end{aligned}$$

11b $$\begin{aligned} \gamma ^*_{11} =\gamma _{11} \end{aligned}$$

must be true.^6^ But, there is little reason to believe Eq. 11 is true, and if it is false it will be difficult to recover $\gamma _{01}$ and $\gamma _{11}$ from the model for $\beta _\mathrm{1j,r}$.

One of two possible conditions can make $r$ sufficient for estimating $\beta _\mathrm{1j}$ unbiasedly. First, if all p$_\mathrm{j}=1.00$, then there is no problem. Of course, if all p$_\mathrm{j}=1.00$, then one has context-representative probability sampling.

Failing this condition, however, one may justify the MLM by assuming:

12 $$\begin{aligned} \beta _{\mathrm{1j,r}} =\beta _{\mathrm{1j,u}} \end{aligned}$$

If Eq. 12 holds, then there is no problem with using only those in group $r$ to estimate the population parameter(s). There is, however, little reason to suspect Eq. 12 to hold in general. Thus, anyone using the MLM must either use context-representative probability samples (i.e., p$_{j}=$ 1.00) or must explain why they believe Eq. 12 holds for the parameters of interest that vary across contexts.

### Macro-level probability sampling

Using the same set-up as above, but presuming all p$$_\mathrm{j}=1.00$$, we now assume that *contexts* are either sampling-reachable ($$s)$$ or sampling-unreachable ($$v)$$. The proportion of contexts in category $$s$$ is q; the proportion of contexts in category $$v$$ is 1$$-$$q. Assignment to category $$s$$ or $$v$$ is not random—the contexts thus differ in unknown but systematic ways, such that category $$s$$ contexts provide little information on the parameter values for category $$v$$ contexts. Thus, sampling from only category $$s$$ contexts is to use a macro-level non-probability sample, for some contexts in the target population are assigned zero chance of selection.

Given Eqs. 1–2, fixed population parameters are estimated without problem, for they simply pass-through the fixed micro-level estimates. However, level-2 population parameters estimated on the sample of macro-level units are necessarily mixtures as in the following:

13 $$\begin{aligned} \gamma _{01} =\text{q}\gamma _{01,\mathrm{s}} +( {\text{1}-\text{q}})\gamma _\mathrm{01,v} \end{aligned}$$

14 $$\begin{aligned} \gamma _{11} =\text{q}\gamma _{{11},\mathrm{s}} +( {\text{1}-\text{q}})\gamma _{\mathrm{11,v}} \end{aligned}$$

which means:

15 $$\begin{aligned} \gamma _\mathrm{01,s} =( {\gamma _{01} -\gamma _\mathrm{01,v} +\text{q}\gamma _\mathrm{01,v}})/\text{q} \end{aligned}$$

16 $$\begin{aligned} \gamma _\mathrm{11,s} =( {\gamma _{{11}} -\gamma _{11,\mathrm{v}} +\text{q}\gamma _{11,\mathrm{v}}})/\text{q} \end{aligned}$$

In other words, using the MLM with macro-level non-probability samples is to use $\gamma _\mathrm{01,s}$ and $\gamma _\mathrm{11,s}$ as if they are $\gamma _{01}$ and $\gamma _{11.}$ The difference between the sought population parameter and the accessible population parameter is a function of q and the associated $\gamma$ parameters, making it difficult to sign the difference.

One of two conditions can make $s$ sufficient for estimating $\gamma _{01}$ and $\gamma _{11}$ unbiasedly. First, if $\mathrm{{q}}=1.00$, i.e., if one has macro-level probability sampling, then there is no problem. Failing this condition, however, one may justify multilevel modeling by assuming:

17 $$\begin{aligned} \gamma _{0\mathrm{1j},\mathrm{s}} =\gamma _{0\mathrm{1j},\mathrm{v}} \end{aligned}$$

18 $$\begin{aligned} \gamma _{\mathrm{11j},\mathrm{s}} =\gamma _{\mathrm{11j},\mathrm{v}} \end{aligned}$$

If Eqs. 17 and 18 hold one may use the MLM with non-probability macro-level samples. There is, however, no general reason to expect Eqs. 17 and 18 to hold. Thus, anyone using the MLM must either use macro-level probability samples (i.e., $\mathrm{{q}}=1.00$) or must explain why they believe Eqs. 17 and 18 hold for the macro-level parameters of interest.

### Two important implications

First, non-FMP samples are not clearly either censored, selected, or truncated samples. Censored, selected, and truncated samples result from probability samples, but non-FMP samples are non-probability samples. Further, in non-FMP samples $Y$ can be observed only for cases that satisfy some variable $Z$, as in selected samples, but $X$ is observed only for cases for which $Y$ is observed, as in truncated samples. The existence of both sample selection and sample truncation problems suggests non-FMP samples may be difficult to repair. The problems posed by censored, selected, and truncated samples are difficult to address, too, but statistical solutions are sought because “better sampling” is often not an option (e.g., no sample design will measure real non-zero wages for those not working for pay). To censored, selected, and truncated samples we must add non-FMP samples in the MLM case, for all four have characteristics that pose challenges for inference. Fortunately, better sample design is a possible solution for non-FMP samples.

Second, analysts sometimes prefer a biased estimator to a less precise unbiased estimator. For example, Hoerl and Kennard (1970) introduce ridge regression which is biased compared to OLS regression but has lower mean-squared error in the presence of multicollinearity. One might argue analoguously that using the MLM on non-FMP samples is better than the alternative.^7^

There are two useful responses to this claim. First, of these observations we must ask, in the non-FMP case, what is the alternative, and what is its cost? The most obvious alternatives to using non-FMP samples are “collect appropriate data” and “ask a question existing data can answer.” Outside of the rare emergency, the only apparent costs to these alternatives is that analysts may have to delay submission for publication until they collect better data or devise a question they can answer, both of which seem to be benefits, not costs, to social science.

Second, the formal analysis indicates that MLMs for non-FMP samples do not simply biasedly estimate the parameter of interest; instead, MLMs for non-FMP samples access the *wrong* population parameter. In ridge regression analysts use a representative sample to estimate $\beta$ with estimable bias; in the non-FMP MLM case analysts use a non-representative sample which estimates a parameter different from the one they seek to target, and the sample design makes the difference between the obtained parameter and the target parameter of unknown sign and magnitude. Thus, tools for scoring the possible trade-off between unbiased imprecise estimators and biased but more precise estimators seem inapplicable, for those tools are designed to aid in comparing different estimators of the *same* parameter, not different parameters.

## Estimation escape? RML and exchangeability as release from sample design demands?

Alas, additional confusion offers two more reasons to continue accepting non-FMP samples. First, one common estimator—restricted maximum likelihood (RML)—does not directly produce estimates of $\beta _\mathrm{1j}$ (Swaminathan and Rogers 2008). This might lead one to presume that the particular macro-level unit in which micro-level units are nested does not matter. However, RML estimates $\beta _\mathrm{1j}$ indirectly. That these estimates still depend on the micro-level data can be demonstrated by simply estimating a model using RML, trading the micro-level data of a few macro-level units with that of other macro-level units, and re-estimating the model. Class A–D coefficients will differ, confirming that micro-level data matter for RML estimation. Moreover, other estimators, such as generalized least squares and full information maximum likelihood (FIML) estimate $\beta _\mathrm{1j}$ directly (Hox 2010, pp. 40–43). Thus, it is incorrect to contend that RML $\beta _\mathrm{1j}$’s are disconnected from their macro-level unit. However, to confirm this expectation simulations will use both FIML and RML estimation.

Second, the MLM is often estimated under an exchangeability or conditional exchangeability assumption, which essentially means that MLMs borrow information from contexts with larger sample size to improve estimates for contexts with sparse data (Braun et al. 1983). Some analysts might interpret the exchangeability assumption as so decoupling level-2 estimates for a given macro-level unit from the level-1 units in that macro-level unit that within-context sample design is irrelevant. However, taking the “decoupled estimates” perspective to this extreme underappreciates the sampling theory that justifies exchangeability. This can best be illustrated by considering conditional exchangeability.

Under conditional exchangeability estimates of macro-level unit $$j$$ are shrunk toward estimates of other macro-level units that are similar. So, considering dichotomous macro-level variable Z, units 1 to $$j-1 $$ for which $$\mathrm{{Z}}=0$$, and units $$j$$ to $$J$$ for which $$\mathrm{{Z}}=1$$, estimates for unit $$j$$ are shrunk more toward estimates for other macro-level units for which $$\mathrm{{Z}}=1$$ (e.g., unit $$j+1)$$ than toward estimates for macro-level units for which $$\mathrm{{Z}}=0$$ (e.g., unit $$j-1)$$. The reasoning is that, owing to the similarity of Z for macro-level unit $$j$$ and units $$j+1$$ to $$J$$, the micro-level relations inside units $$j+1$$ to $$J$$ are presumed to contain more relevant information on the micro-level relations inside unit $$j$$ than do dissimilar macro-level units $$1$$ to $$j-1$$. Yet, this presumption requires that relations inside units $$j+1$$ to $$J$$ be unbiasedly estimated. If the relations inside these units are estimated using micro-level context unrepresentative samples, then systematic error attends the estimates, and, more important, being non-probability samples, extrapolation (e.g., borrowing information) from them is rendered indefensible. Given this chain of reasoning, it seems difficult to claim that exchangeability obviates FMP sampling.^8^

The only difference between exchangeability and conditional exchangeability is the former borrows from all macro-level units according only to the precision of their estimates, while the latter does so weighted also by the proximity of the macro-level contexts to each other in multidimensional space defined by the macro-level variables in the model. Either way, sampling theory as discussed here indicates that exchangeability requires probability sampling.

The textual and formal explications suggest costs of non-probability sampling for the MLM. What does the didactic MLM literature say on these issues?

## The multilevel model in didactic perspective

In the late 1980s multilevel modeling became increasingly feasible with increases in computing power and speed (Fuchs 2001) that facilitated implementation of iterative estimation techniques, such as the EM algorithm (Turnbull 1976; Dempster et al. 1977) for multivariate analyses. Although some of the journal literature explicitly noted the necessity of probability samples at all levels (e.g., Aitkin and Longford 1986, pp. 12–13; Longford 1987, p. 819), as model estimation difficulties declined descriptions of the MLM diffused from the journals to the textbook literature. However, sample imperatives did not diffuse as well.

Bryk and Raudenbush (1992) offered one of the earliest, most accessible textbook treatments. The text understandably emphasized the complexities of model specification and interpretation for an audience unfamiliar with the framework. Still, the resulting silence on MLM sample requirements affected analysts’ understanding of the MLM.

Although many MLM textbooks ignore sampling (e.g., Longford 1993; Kreft and de Leeuw 1998; Raudenbush and Bryk 2002; Goldstein 2003; Pinheiro and Bates 2004; Twisk 2006; Bickel 2007; Gelman and Hill 2007; McCulloch et al. 2008; Rabe-Hesketh and Skrondal 2008; Heck and Thomas 2009; Heck et al. 2010), a few do address sampling. Snijders and Bosker (1999) is fairly explicit, distinguishing between context as nuisance versus context as substantive interest, outlining the operation and cost-effectiveness of multi-stage sampling, and contending multilevel modeling is necessary for such samples. Indeed, Snijders and Bosker provide an enlightening discussion of sampling, as far as it goes. However, at the precipice of discussing whether MLM requires context-representative micro-level probability sampling the treatment veers off into other, important, but different territory, leaving the question unasked and, thus, unanswered.

Textbook authors might regard FMP sampling as obviously required. Yet, they also recognize researchers may use secondary data (Snijders and Bosker 1999, p. 140); there is no guarantee secondary data will be FMP samples. Indeed, the rising tendency to attach geocode data to datasets may make use of non-FMP samples for multilevel modeling proportionately more common than otherwise. The likelihood analysts will use non-FMP samples is driven even higher by failure to explicitly inform analysts about the issue.

Indeed, Luke (2004), in what is colloquially known as the little green Sage book series, legitimates non-FMP samples, stating: “the minimal data requirement for multilevel analysis is a dependent variable and a link to a level-2 unit. However, in most cases, the datasets will also include a variety of level-1 and level-2 predictors. (Luke 2004, p. 16).” Hox (1995) also accepts non-probability sampling, writing “Of course in real research one may have a convenience sample at either level” (Hox 1995, p. 1), a position the opposite of that argued here. Thus, some published advice on the MLM explicitly indicates that FMP samples are unnecessary.

Since popularization of the model the MLM technical literature has continued to treat sampling even though sampling is largely ignored in textbook treatments. Yet, the technical literature has focused on determining the implications of various sample size patterns (e.g., Reise and Duan 2003; Moerbeek et al. 2008). This research has established the trade-offs involved in sampling many macro-level contexts and few micro-level units versus the opposite design. This work is important. Still, there are other important sampling issues.

Finally, some might see MLM equations as indicating sample design demands, viewing theorized error distributions as invoking the central limit theorem, thus implying probability sampling, therefore warranting the claim that the equations clearly convey sample requirements. Such a chain of claims ignores that theorized error distributions are oft-violated assumptions whose violation may not render data inappropriate for inference (Hubbard 1978). Further, although they may be related, error distribution assumptions are not sample design imperatives.

In general, interpreting any equation requires supplementary information, not only the content of the equations. For example, nothing in Eq. 1–2 stipulates that $\text{E}(\hat{\beta}_{1\mathrm{j}} )=\beta _{1\mathrm{j}}$. In fact, the expected value of $\hat{\beta}_{1\mathrm{j}}$ depends on many factors. As de Leeuw and Kreft (1995) note, one must distinguish between model, technique, algorithm, and computer program, and to that list we could add “sample design.” The equations only reference the model, yet model, technique, algorithm, program, and sample design collectively constitute a practical estimator and thus determine its properties. It is easy to fall into assuming that an equation conveys the necessity of appropriate sampling, but, even if so, one must fill the category “appropriate sampling” with content to move from the chalkboard equations of a model to its appropriate use on real data. In that connection, it is clear that one can estimate a model using inappropriate data, such as, for example, convenience sampled data, and calculate coefficient estimates and standard errors. We should expect, however, such estimates to be erroneous and inference to be indefensible even though the formulas matched the equations expressed in chalk. That is our expectation in the single-level case, and nothing in the MLM should change that expectation. Still, some analysts seem to support use of the MLM on such samples (e.g., Hox 1995; Luke 2004).

This is all to say that analysts may have been insufficiently sensitive to the data demands of the MLM, thereby explaining both how some could estimate the MLM while failing to attend to high sample design requirements, and why the necessity of FMP samples for MLMs need be explicitly established.

Of course, one might imagine that perhaps the issue is neglected because the problem is rare. What remains to be determined, therefore, is whether probability samples are commonly transformed into non-probability samples for multilevel modeling and, if so, to assess empirically whether the transformation undermines inference. The next section addresses the first issue, while the remainder of the text addresses the second.

## Inconvenient datasets: workhorse datasets as non-probability samples in the multilevel context

The value of probability samples for inference is well-established (e.g., Yates 1946). Thus, official data collection efforts either draw probability samples or conduct censuses. Official or statistical analysis of non-probability samples has been relegated to exploratory research (e.g., Banyard et al. 2001), emergencies (e.g., Başoğlu et al. 2002), studies of hidden populations (e.g., Bluthenthal et al. 1998), and combinations thereof (e.g., Centers for Disease Control 1981). While mindful of such uses, the higher value of probability samples is accepted by researchers endeavoring to discover ways to make non-probability samples approximate the properties of probability samples (e.g., Salganik and Heckathorn 2004).

Most large-scale data collection is designed to produce probability samples that represent larger aggregations, such as the nation or region, not lower levels of aggregation, such as states, cities, or neighborhoods. Yet, the published empirical literature contains multiple studies using data designed to represent larger aggregations to estimate MLMs for other geo-social contexts. Briefly considering that literature suggests the widespread nature of the problem.

### Common complex sample designs and the MLM

Very complex sample designs, even when devised to allow multilevel investigation, can still be insufficient for some multilevel research. The National Education Longitudinal Study of 1988 (NELS) is a multi-wave study of students in schools. Base-year data collection surveyed 24,599 8th graders attending 1,052 schools. Additional data collection occurred in 1990, 1992, 1994, and 2000. Early waves of NELS data were released just as software allowing appropriate MLM estimation was becoming widely available, timing which may have spurred use of the model with NELS. Yet, beyond the base year NELS appears inappropriate for multilevel modeling, because NELS becomes a non-probability sample when students’ 10th or 12th grade schools are used as contexts.

Two key reasons underlie this limitation. First, NELS base year eighth graders were sampled to represent peers in their middle school, making it appropriate to draw inferences about each 8th grade school using the sampled students for the school. However, 2–4 years later the NELS students do not constitute a probability sample for their particular high school, because many high schools are fed by more than one eighth grade school, and eighth grade schools were not sampled to represent feeder-school patterns—that is, eighth grade schools were neither sampled in relation to particular high schools to which they fed, nor in proportion to the likelihood their students would be in any given high school, nor was there sufficient knowledge to weight the data afterwards to account for feeder school patterns. Thus, when sampled students show up at high school, they fail to represent their high school peers who come from other, non-sample, eighth grade schools.^9^ $$^{,}$$ 10

To concretize this contention, we know that 8th grade schools differ on a host of characteristics (e.g., poverty, racial composition, amenities, parents’ mean level of education). Further, we know that high schools, being bigger, will contain students from multiple eighth grade schools. Given eighth-grade school variation, it is conceiveable that in a given high school the parameter relating the effect of parental education on the likelihood of taking calculus is one value for students who entered from eighth-grade school $j=1$, partly reflecting the view faculty have of that school, and *different* values for students who entered from schools $j=2$ to $J$, partly reflecting the different views faculty have of those other schools; in other words, we posit that $\beta _\mathrm{1j,r}\ne \beta _\mathrm{1j,u}$. If this is true, then the NELS design will mis-estimate the high school-specific slope of parental education on high-level course-taking, as well as the correlates of that slope (i.e., the role of high school characteristics in determining the power of parental status characteristics on course assignment). Thus, one cannot use measured within-high school *statistical* relationships to discern the within-high school *social* relationships unless one assumes either that the $J$ feeder schools for each high school, $h,$ are indistinguishable on all relevant observables and unobservables, or that the chosen feeder school(s) had been probability sampled from the $J$ feeder schools for each high school. We know both assumptions are untrue for NELS. Yet, one of those heroic assumptions is required because they are the only ways that high school students from sampled eighth grade schools can be seen to represent their high school peers who attended other eighth grade schools given the NELS sample design.

Second, NELS high schools are not a probability sample of U.S. high schools. The NELS 8th grade schools are a probability sample of 8th grade schools, but two years later the schools these students attend are not a probability sample of U.S. high schools (Ingels et al. 1994).

Thus, using the MLM with NELS follow-up waves is to estimate statistical models on context-unrepresentative micro-level samples of students for a non-probability sample of high schools. Resulting MLM estimates should be both biased and ungeneralizable.

NELS project staff responded to these limitations by producing a supplement, the High School Effectiveness Study (HSES). HSES students form a probability sample within each school and sampled schools are a probability sample for large urban and suburban districts. This is a useful response, but one cannot generalize to the nation using HSES (Scott et al. 1996).

Some researchers have recognized the limits and attempted to address the problem while still using NELS for post-base-year MLM research. For example, Lee and Smith (1995) construct and use post hoc weights. They note that although National Center for Education Statistics (NCES) usually provides school weights, NCES did not provide school weights for follow-ups of NELS. Perhaps the reason NCES did not provide the weights is that they could not determine weights that would make the non-probability sample of high schools a probability sample. Although Lee and Smith (1995) note that “The high schools were not selected in the NELS sampling frame, but were selected by NELS students” they appear to disregard the implication, continuing, “Since [sic] our research questions focused on variation among [high] schools, we needed school weights. (Lee and Smith 1995, p. 247).” Needing high school weights, Lee and Smith attempted to produce them.

To evaluate their success (Lee and Smith 1995, p. 264) then used the same method to construct weights for the eighth grade school, after which they estimated MLMs first on unweighted eighth grade data, then on eighth grade data weighted using NCES weights, and then on eighth grade data weighted using Lee and Smith (L&S) weights. They report that L&S eighth-grade weighted results are closer to NCES weighted results than to unweighted results, and thus they conclude their L&S *high school* weights are also appropriate. This is a conscientious exercise, but, alas, it does not address the question of whether the high school weights make NELS an FMP sample after the base year. The L&S eighth-grade weights served to re-weight a probability sample, whereas the L&S high school weights attempt to transform a *non*-probability sample into a probability sample. Comparisons of results of different weighting schemes using a probability sample do not prove that L&S weighting transforms a nonprobability sample into a probability sample.

Lee and Smith (1995) recognized difficulty and attempted to address it. Other researchers appear unaware of the issue, perhaps believing the sample is appropriate for the MLM because probablity sampling was used for base-year NELS. For example, Roscigno (1998) estimates MLMs on NELS high school students to assess the role of institutional factors in reproducing racial inequality, reporting that “NELS represents a nationally representative sample of U.S. high school students ....Sampling was first conducted at the school level and then at the student level within schools. I focus on and use the first follow-up (1990) of the study, in which all respondents are tenth graders (Roscigno 1998, pp.1036–1037).” Roscigno (1998) describes the base-year sample design, and appears to assume follow-up data are also appropriate for the MLM. Unfortunately, despite the base year design, the follow-up NELS data are not FMP samples. Inadvertent use of inappropriate samples is exactly what can happen if researchers are not apprised of the high data demands of the MLM posited here.

Other researchers appear to ignore sample design. For example, in researching the role of disciplinary climate and practices on student achievement and safety (Arum 2003) uses NELS data and multilevel modeling, sometimes nesting students in high schools, sometimes nesting students in states. Although neither context appears appropriate with NELS, the state linkage is most questionable. Nothing in the NELS codebooks suggests state-level inference was ever appropriate; indeed, the expansion of state-level National Assessment of Educational Progress (State-NAEP) was spurred in part by this limitation of the NCES longitudinal studies program (Pellegrino et al. 1999, especially pp. 36–37). Using states as contexts with NELS is justified, however, if one believes a link between level-1 and level-2 units is all that is required to usefully estimate the MLM, as Luke (2004) contends and Hox (1995) suggests.

### The dangers of simpler designs

Inappropriate data for the MLM can be produced by much simpler designs. For example, General Social Survey (GSS) cross-sectional data have been collected annually or bi-annually since 1972. The sample design has changed but, with the exception of the extension to cover non-English speakers, the target population has remained the same. The design allows inferences to (English-speaking) adults living in non-institutional, non-group quarters households in the continental U.S. The design stratifies primary sampling units by region, race, and age.

Geocode data is now available for GSS respondents. However, GSS is designed only to generalize to the nation, region, or counties/SMSAs. Respondents are not sampled to be representative of the state, census tract, or any other geographic dimension of residence.^11^ Yet, some use GSS geocode data as if respondents do form probability samples for such contexts.

So, for example, Berkman and Plutzer (2009) allocate GSS respondents to states and use the MLM in their study of public opinion on the coverage of evolution in schools. They find public opinion towards evolution matters for whether evolution is taught, but that gross state product per capita and the administrative capacity of the state education office do not matter. Although the finding is plausible, it is also possible that the estimates overstate the role of public opinion; the true model might not include public opinion, either. Thus, although the finding may eventually be confirmed, this work should be set aside.

The GSS is but one example; similar features characterize other workhorse datasets as well. For example, the National Longitudinal Study of Adolescent Health (AdHealth) sampled schools and, within schools, sampled students. The existence of private schools, school choice plans, home schooling, grade retention practices, grade acceleration practices, varying maximum compulsory education ages, juvenile justice policies, and student and adult action around the above all mean that to sample within schools is not to sample within neighborhoods. Indeed, the disjuncture between schools and neighborhoods is a complex function of those processes and more, and varies nonrandomly. Thus, schools (and the students of them) are distinct from neighborhoods (and the children in them). Yet, analysts have used AdHealth to study neighborhood effects with the MLM.

For example, in a series of papers Harding (2007, 2008, 2009, 2011) uses AdHealth geocoded census tract data to study how neighborhood characteristics, theorized as cultural context, matter. Among other findings, Harding reports that disadvantaged neighborhoods have more cultural heterogeneity and in such neighborhoods adolescents’ ideologies are less predictive of their sexual behavior. While plausible, it is also possible that disadvantaged neighborhoods have less cultural heterogeneity and in such neighborhoods adolescents’ ideologies may be more predictive of their sexual behavior. Indeed, although each conclusion in the series of papers is plausible, so is its opposite. As AdHealth students are a non-probability sample of neighborhood children, representing their schoolmates, not their neighbors, inferences based on neighborhoods is, I argue, biased to an unknown degree and in an unknown direction. If this claim is correct, the findings from these papers should be disregarded,^12^ for their inclusion in the research record threatens to direct research down unproductive lines (Lucas 2013a). If findings from such studies were erased from the record, ruled out of order from consideration by the academic “jury,” what directions for research would no longer have substantive warrant? The difficulty of “unringing a bell” suggests that serious damage can be done to social science understanding and research when biased data becomes an accepted norm for empirical research.

The National Longitudinal Survey of Youth 1979, the Survey of Income and Program Participation, and the Panel Study of Income Dynamics are among the datasets now available with attached geocode data. Geocode data may have value for other models, and used differently–as in Fuller’s (2008) wage growth models—has value in the MLM.^13^ But, enamored of the MLM, analysts are moving to exploit geocode data by assigning respondents to geo-social contexts geocoding allows but for which respondents are not representative, because the analysts are either unaware of or undeterred by the MLM requirements sampling theory implies. The findings they produce thereby are perhaps seriously misleading.

### On inconvenient datasets

I contend that many datasets do not allow inference for level-2 MLM parameters for many geo-social levels of analysis. Yet, the examples above indicate that some published empirical research implicitly denies this contention. Indeed, the list of MLM analyses using these and other datasets to study geo-social contexts to which the sample was not designed to generalize is long; space considerations preclude provision of such a list here. Thus, the current condition is that such analyses can be published, and at least some methodologists have contended that such analyses are appropriate. Yet, I argue, basic sampling theory suggests otherwise. In response, I turn to Monte Carlo simulation to resolve the issue.

## Monte Carlo simulations

I conduct two studies. In the first students are sampled in simulated middle schools, but the multilevel analysis investigates high school effects. This design is similar to the NELS design. The second study employs a simpler design; persons are sampled via one geo-social contextual dimension, but inference is to some other geo-social dimension which is not the basis of sampling. This design resembles datasets, such as GSS and AdHealth, that provide geocode data on contexts (e.g., census tracts) to which the sample was not designed to generalize. Table 6 in Appendix contains the correlation matrices for both datasets.

There are at least two ways to conduct monte carlo simulations. One popular approach (e.g., Boomsma 1987; Stolzenberg and Relles 1990) identifies key parameters that determine the population, sample design, and/or measurement, varies those parameters systematically, and investigates the performance of an estimator as the parameters in the simulation change. Such simulations map the robustness of the estimator under different conditions.

An alternative approach constructs plausible conditions and tests the performance of the method under these conditions alone. This approach attends less to the forest of possibilities, focusing instead on trees known to exist. Because it may entail thousands of analyses of one population, it is a monte carlo study. The simulations below are of this type.

The reason for this strategy is that there is an almost inexhaustible set of ways one can produce a non-probability sample from a probability sample, and no clear small set of parameters to capture that variety. In the case of simulations studying selection bias effects on an estimator, for example, one could vary only three parameters–the proportion of selected cases, the correlation between selection and the outcome, and the correlation between selection and the covariates (e.g., Stolzenberg and Relles 1990). Even these three can result in a startlingly high number of possibilities. However, taking the formal analysis here as an example, one would need to vary p$_\mathrm{j},\,\beta _\mathrm{ij,r},\,\beta _\mathrm{ij,u},\,\gamma _{10},\,\gamma _{11}$. But, what does this really mean? p$_\mathrm{j}$ is not one value, it is a vector of values $(\mathbf p _\mathrm{j})$, one p$_\mathrm{j}$ per contextual unit. Each vector $\mathbf p _\mathrm{j}$ represents one population from which the simulation would sample. And, the same goes for the $\beta$’s. The different populations should have different means and variances for p$_\mathrm{j}$ and the $\beta$’s. Without this complexity, the simulation will only indicate how the MLM works for one set of p$_\mathrm{j}$ and $\beta$’s, an outcome little different than the alternative strategy provides. Yet, the complexity continues, for one would need to vary the pattern of values of independent variables across macro-context samples (to address exchangeability), and the *pattern* of macro-context sample sizes (e.g., all equal-sized, one big outlier, one small outlier, multiple big outliers, multiple small outliers, one big and one small outlier, one big outlier with multiple small outliers, multiple big outliers with one small outlier, multiple big and small outliers). One could simplify the simulation, but then the simulation could be dismissed as “unrealistic.”

Owing to this complexity, I eschew this approach, opting instead for matching plausible existing sample designs, reasoning that if the method fails under conditions similar to those of known designs, then the case for caution will have been made.

### Study 1—design

For study 1 I constructed a population of approximately 3.9 million middle school students. Each student was assigned to one of the 11,200 middle schools. I used the concept of *feeder school*, a middle school whose students are likely to attend a particular high school. There were 4,000 high schools; four hundred had one feeder school, 1,200 had two, 1,200 had three, and 1,200 had four. Each middle school was randomly assigned an enrollment of 300–400 8th graders.

A feeder school need not feed most of its students to its destination high school. Indeed, Schiller (1999) reports that fewer than 10 % of NELS 8th graders attended the same high school as the rest of their 8th grade classmates. Thus, although the average student attended a high school with 62 % of their 8th grade classmates, the standard deviation of this estimate was 34 % points, indicating considerable scatter from some middle schools.

I randomly assigned a “feeding probability” of 30–100 % to each middle school which reflected the likelihood its students would attend the destination high school, and randomly assigned an attrit probability to each student reflecting the likelihood that the student would not attend the destination high school. If a student’s attrit probability exceeds their school’s feeding probability, then the student is provisionally classified as not enrolling in the high school; otherwise, the student transitioned to the destination school. On this basis 34 % of the students did not follow their peers.

I then randomly assigned each high school a uniformly-distributed interval-level variable, Z$_{1}$, which could be regarded as an indicator of school quality or teaching strategies at the school. The uniform distribution was used to assure thick representation in the population throughout the full distribution of Z$_{1}$, which increases the chance that each Monte Carlo sample would also have representation throughout the full distribution of Z$_{1}$.

Each middle school and each student was randomly and independently assigned to one of 7 types. We might regard these types as indicators of type of instructional strategies a school might employ and a student might need. A variable reflecting whether the student and the school matched types was then constructed. I then constructed X$_{1}$ at the student-level, a function of the type of school, the existence of a match between the student-type and the school-type, and a random variable. Then, X$_{2}$ was constructed as a function of X$_{1}$ and a random component.^14^

The student-type school-type match variable also figured in the final construction of the dichotomous indicator of students’ attrition. I modified the provisional dichotomous variable such that any student whose type matched the school type did not attrit; I reasoned that students well-matched with the middle-school will be more likely to stay on the “expected” trajectory.^15^ After this modification 29 % of students attritted; thus, 71 % of students attended high school with middle school peers, a figure somewhat larger than the 62 % reported for NELS (Schiller 1999). Thus, if scatter increases bias, then Study 1 data may be less likely to produce biased estimates than NELS.

Two random student-level errors ($\varepsilon _\mathrm{ij1}$ and $\varepsilon _\mathrm{ij2})$ and two random high-school level errors ($\delta _\mathrm{j1}$ and $\delta _\mathrm{j2})$ were used to construct two dependent variables, as follows:

19 $$\begin{aligned} \text{Y}_{\mathrm{1ij}} = 20+.35 \text{X}_{\mathrm{1ij}} +.45 \text{X}_{\mathrm{2ij}} +.20\text{Z}_{\mathrm{1j}} +\varepsilon _{\mathrm{ij1}} +\delta _{\mathrm{j1}} \,\text{and}, \end{aligned}$$

20 $$\begin{aligned} \text{Y}_{\mathrm{2ij}} = 40+.40\text{X}_{\mathrm{1ij}} +.20\text{X}_{\mathrm{1ij}} \text{Z}_{\mathrm{1j}} +\text{X}_{\mathrm{1ij}} \delta _{\mathrm{j2}} +.50\text{X}_{\mathrm{2ij}} +\varepsilon _{\mathrm{ij2}}, \end{aligned}$$

which can be analyzed in means-as-outcomes and slopes-as-outcomes models.

Each replication probability samples 250 middle schools and 30 students within each middle school. Sampled students who attrit between middle and high school are dropped from the high school sample, similar to as in NELS. I obtained 5,000 samples and for each sample estimated four models for each dependent variable: (1) a MLM estimated using FIML, (2) a MLM estimated using RML, (3) a quasi-weighted multilevel model (QWMLM), and (4) an OLS regression model with Huber–White standard errors (HWLS).

I estimate the HWLS model because analysts often use this approach to adjust standard errors for clustering in the sample (Froot 1989). However, it is not clear that HWLS coefficients would be unbiased when estimated on inadvertant non-probability sample data. Thus, we check this possibility. QWMLM employs weights for students and schools, weighting students under the assumption that sampled students represent their high school peers, and weighting schools as if they represent other high schools even though the sample design was not so arranged. As Winship and Radbill (1994) show, weighting often introduces biases in regression models. Still, some scholars have attempted to produce weights to facilitate using non-probability data with the MLM. Thus, the QWMLM indicates what one might learn using post hoc weights for all levels when one lacks FMP samples.

### Study 1—results

Table 2 contains the results of a means-as-outcomes analysis. The first fact to notice is that restricted maximum likelihood estimates are exactly the same as full information maximum likelihood estimates. Thus, whatever we find for the latter pertains to the former.

**Table 2:** Study 1 Monte Carlo means-as-outcomes multilevel models and “Huber–White” OLS results of parameter estimates for Y1, 5,000 replications

Population parameter				RML estimation		Full information maximum likelihood estimation				OLS estimation	
				Multilevel		Multilevel		Quasi-weighted MLM		Huber–White	
Labels	Level	Class	Value	Mean	SD	Mean	SD	Mean	SD	Mean	SD
$\beta _{0j}$ for constant (=$\lambda _{00})$	2	D	20.00	20.028	.220	20.028	.220	20.054	.235	20.058	.237
Standard error for $\beta _{0}$				.220	.004	.220	.004	.976	.053	.238	.014
$\lambda _{10}$ for Z$_{1}$	2	B	.200	.140	.176	.140	.176	.140	.176	.140	.181
Standard error for $\lambda _{10}$				.180	.009	.180	.009	1.640	.088	.186	.015
$\beta _{1}$ for X$_{1}$(=$\lambda _{01})$	1	F	.350	.351	.027	.351	.027	.350	.029	.353	.034
Standard error for $\beta _{1}$				.026	.001	.026	.001	.026	.0008	.034	.003
$\beta _{2}$ for X$_{2}$(=$\lambda _{02})$	1	F	.450	.450	.004	.450	.004	.450	.004	.450	.004
Standard error for $\beta _{2}$				.004	.00006	.004	.00001	.003	.0001	.004	.0002

With respect to both, class F coefficients appear unbiasedly estimated. If one is interested in level-1 coefficients, even if one lacks FMP samples, there appears no cost to using the multilevel model, just as theorized. However, despite the posited requirements for the class D coefficient, it is overestimated by less than 2/100 of 1 %, a discrepancy that might easily be due to rounding. The biases for the QWMLM and HWLS class D coefficients are larger but on the same order.

Typically, however, the means-as-outcomes model is estimated because of an interest in the class B coefficient, the coefficient describing the association between macro-level variables and the context-specific mean. And, the MLM class B coefficient—a level-2 coefficient—is demonstrably biased. The population parameter is .200, but the mean maximum likelihood estimate is .140, an underestimate of 30 %. QWMLM and HWLS estimates are equally biased.

These results indicate that although the class D coefficient may not be biased, the coefficients which are usually the motivation for means-as-outcomes models are biased when one has a context-unrepresentative micro-level sample and lacks a macro-level probability sample. Further, weighting does not address the problem.

Table 3 contains results of models for Y$_{2}$ under the same sample design.^16^ Restricted maximum likelihood estimates equal the maximum likelihood estimates.

**Table 3:** Study 1 Monte Carlo slopes-as-outcomes multilevel models and “Huber–White” OLS results of parameter estimates for Y$_{2}$, 5000 replications

Population parameter				RML estimation		Full information maximum likelihood estimation				OLS estimation	
				Multilevel		Multilevel		Quasi-weighted MLM		Huber–White	
Labels	Level	Class	Value	Mean	SD	Mean	SD	Mean	SD	Mean	SD
$\beta _{0}$ for constant (=$\gamma _{00})$	1	E	40.000	40.006	.256	40.006	.256	40.012	.277	40.002	.263
Standard error for $\beta _{0}$				.257	.004	.257	.004	.254	.004	.263	.013
$\beta _{1j}$ for X$_{1}$(=$\gamma _{01})$	2	C	.400	.358	.091	.358	.091	.364	.113	.357	.102
Standard error for $\beta _{1}$				.094	.005	.093	.005	.997	.085	.108	.013
$\gamma _{11}$ for Z$_{1}$	2	A	.200	.275	.153	.275	.153	.263	.187	.284	.168
Standard error for $\beta _{1}$				.155	.009	.154	.009	1.707	.143	.168	.019
$\beta _{2}$ for X$_{2}$(=$\gamma _{02})$	1	F	.500	.500	.005	.500	.005	.500	.005	.500	.005
Standard error for $\beta _{2}$				.005	.00008	.005	.00008	.005	.00008	.005	.00024

Looking at these estimates, it appears that level-1 parameter estimates are either spot on or so negligibly different from their population parameters the difference could be due to rounding. In contrast, the estimates of the level-2 coefficients are demonstrably biased. The class C coefficient is a 10.5 % underestimate; an almost equal-sized discrepancy is obtained with the HWLS estimate, while the QWMLM approach produces a 9 % underestimate. Thus, all four approaches estimate the class C coefficient with bias.

The bias is larger for the class A coefficient, the association between the macro-level factor and the slope for a micro-level variable. While HWLS overestimates by 42 %, and QWMLM is less biased, overestimating by 31 %, maximum likelihood methods overestimate the population parameter by 37.5 %, even though the population correlation of non-attrition with Y$_{2}$ is .036. Despite the minimal correlation, large biases for MLM parameters are obtained.

Note that for the class A coefficient the quasi-weighted MLM results are marginally less biased than the maximum likelihood estimates. Yet, QWMLM results are still seriously biased. Thus, it appears weighting, at least as employed here, does not solve the sample design problem.

### Study 2—design

I constructed 144 primary sampling units (PSUs). PSUs are of equal size, and fall into 2 strata based on the proportion minority. Each PSU is assigned a random score that indicates its likelihood of having minorities residing in the PSU. In total there are 12 minority PSUs and 132 white PSUs. Each PSU contains 100,000 persons, and each person is assigned a race. Every PSU has some minorities and whites.

PSUs are not of substantive interest. There are, instead, 50 states that serve as geo-political divisions. States vary in size, and this is reflected in the number of PSUs in the state. The largest state has 6 PSUs, the smallest states have only 1. There is no effort to represent every state in a given sample, nor to assure that the cases sampled represent those in their state.

For each person two micro-level random variables (X$$_{1}$$ and X$$_{2})$$ are produced which are in part a function of race and the PSU in which the case is located. Minorities have lower average levels of each variable. Further, a random, uniformly-distributed, state-level variable, Z$$_{1}$$, is assigned to each state; the variable is uniformly distributed to increase the chance that a wide-range of values for the variable will appear in any sample. And, two random person-level errors ($$\varepsilon _\mathrm{ij1}$$ and $$\varepsilon _\mathrm{ij2})$$ and two random state-level errors ($$\delta _\mathrm{j1}$$ and $$\delta _\mathrm{j2})$$ were produced. Then, two dependent variables were constructed as follows:

21 $$\begin{aligned} \text{Y}_{\mathrm{1ij}} =10+.3 \text{X}_{\mathrm{1ij}} +.\text{2X}_{\mathrm{2ij}} +.\text{25Z}_{\mathrm{1j}} -.1\,\text{Minority}_{\mathrm{ij}} +\varepsilon _{\mathrm{ij1}} +\delta _{\mathrm{j1}} \,\text{and}, \end{aligned}$$

22 $$\begin{aligned} \text{Y}_{\mathrm{2ij}} =15+.2 \text{X}_{\mathrm{1ij}} +.\text{3X}_{\mathrm{1ij}} \text{Z}_{\mathrm{1j}} +\text{X}_{\mathrm{1ij}} \delta _{\mathrm{j2}} +.\text{6X}_{\mathrm{2ij}} -.05 \text{Minority}_{\mathrm{ij}} +\varepsilon _{\mathrm{ij2}} \end{aligned}$$

such that Y$_{1}$ might be analyzed in a means-as-outcomes approach, while Y$_{2}$ might be analyzed in a slopes-as-outcomes approach.

Note that for each dependent variable the PSU effect on Y is channeled completely through X$_{1}$ and X$_{2}$, reflecting the idea that those in the same PSU are somewhat similar in comparison to those in some other PSU. The state-level variable Z$_{1}$, however, is not a function of the PSU. Instead Z$_{1}$, as a state characteristic, applies equally to every micro-level unit within the state regardless of the PSU in which the unit is nested.

All told, there are 14.4 million persons in the PSUs, of which 29.8 % are minority. Fully 91.8 % of those in the minority PSUs are minority; in contrast, 24.1 % of those in the white PSUs are minority. Each Monte Carlo replication probability samples one minority PSU and 11 nonminority PSUs and in each PSU 1,000 persons are probability sampled.

### Study 2—results

Table 4 contains results of 5,000 replications of the means-as-outcomes model.^17^ Both FIML and restricted maximum likelihood level-1 coefficients (class F coefficients) are estimated without bias. In contrast, all level-2 coefficients have bias. For the FIML MLM the intercept (class D) is slightly underestimated, and the class B coefficient is overestimated by over 5 %. Thus, the key multilevel parameter, estimated using FIML, is biased. HWLS and RML fare worse.

**Table 4:** Study 2 Monte Carlo means-as-outcomes multilevel models and “Huber–White” OLS results of parameter estimates for Y$_{1}$, 5000 replications

Population parameter				RML MLM		FIML MLM		“Huber–White” LS	
Labels	Level	Class	Value	Mean	SD	Mean	SD	Mean	SD
$\beta _{0j}$ for constant (=$\lambda _{00})$	2	D	10.00	9.841	.312	9.911	.288	9.915	.313
Standard error for $\beta _{0}$				.318	.084	.303	.080	.271	.091
$\lambda _{10}$ for Z$_{1}$	2	B	.25	.277	.329	.263	.306	.265	.311
Standard error for $\lambda _{10}$				.316	.116	.307	.112	.231	.101
$\beta _{1}$ for X$_{1}$(=$\lambda _{01})$	1	F	.30	.300	.008	.300	.008	.352	.107
Standard error for $\beta _{1}$				.008	.0004	.008	.0004	.084	.031
$\beta _{2}$ for X$_{2}$(=$\lambda _{02})$	1	F	.20	.200	.008	.200	.079	.189	.104
Standard error for $\beta _{2}$				.008	.0004	.008	.0003	.083	.028
$\beta _\mathrm{Minority}$	1	F	-.10	-.0003	.031	-.100	.032	-.113	.397
Standard error for $\beta _\mathrm{Minority}$				.031	.003	.031	.004	.320	.112

RML estimation produces a bias of 10.8 % for the class D coefficient, more than twice the bias obtained with FIML estimation, a result that contradicts the view that RML estimation liberates coefficients from requirements for FMP sampling. Further, RML estimates of the level-1 coefficient for minority—the dimension along which the sample is stratified—is underestimated by over 99 %.

HWLS also fares poorly, for all level-1 and level-2 HWLS coefficients are biased. The class B coefficient is overestimated by 6 %, and level-1 coefficients are overestimated by up to 17.33 % and underestimated by up to 5.5 %.

Summarizing the findings with respect to the class D coefficient, however, Table 4 findings mean that in general the class D coefficient is biased when estimated using non-FMP samples, just as theorized.

Table 5 extends the analysis to the slopes-as-outcomes model.^18^ Considering the FIML results, all level-1 estimates are unbiased or so very slightly different from the true value that rounding could be the cause. Yet, all level-2 estimates are biased. The class A coefficient is underestimated by 11 %, and the class C coefficient is underestimated by 28.5 %.

**Table 5:** Study 2 Monte Carlo slopes-as-outcomes multilevel models and “Huber–White” OLS results of parameter estimates for Y$_{2}$, 5000 replications

Population parameter				RML MLM		FIML MLM		“Huber–White” OLS	
Labels	Level	Class	Value	Mean	SD	Mean	SD	Mean	SD
$\beta _{0}$ for constant (=$\gamma _{00})$	1	E	15.00	15.000	.013	15.000	.013	15.054	.347
Standard error for $\beta _{0}$				.013	.001	.013	.001	.267	.111
$\beta _{1_j}$ for X$_{1}$(=$\gamma _{01})$	2	C	.20	.171	.388	.143	.380	.079	.426
Standard error for $\beta _{1}$				.417	.111	.421	.104	.382	.126
$\gamma _{11}$ for Z$_{1}$	2	A	.30	.318	.354	.267	.348	.281	.399
Standard error for $\gamma _{11}$				.418	.163	.428	.157	.314	.157
$\beta _{2}$ for X$_{2}$(=$\gamma _{02})$	1	F	.60	.600	.007	.600	.007	.585	.146
Standard error for $\beta _{2}$				.007	.001	.013	.001	.110	.048
$\beta _\mathrm{Minority}$	1	F	-.05	-.001	.027	-.049	.027	-.096	.750
Standard error for $\beta _\mathrm{Minority}$				.027	.003	.027	.002	.547	.238

Restricted maximum likelihood fares better, producing an overestimate of 6 % and an underestimate of 14.5 % for the class A and class C coefficients, respectively. Yet, RML estimation produces an estimate for the level-1 coefficient for minority that is a 98 % underestimate of its true value. Considering both variants of maximum likelihood estimation, it appears that the MLM estimated on such data produces biased estimates of key parameters.

Further, again, HWLS does not solve the problem. All HWLS coefficients are biased, and, notably, the class C coefficient—a level-2 coefficient—is underestimated by over 60 %.

Thus, Tables 4 and 5 indicate that analysts interested in level-2 parameters should avoid context-unrepresentative micro-level samples. And, the analysis suggests that analysts using stratified data might be especially concerned about parameter estimates for the variables along which the sample design stratifies. Notably, the findings mean that geocode data offers little value for multilevel modeling of most geo-social levels.

Examining all analyses, MLM estimates are sometimes positively and sometimes negatively biased. Thus, the bias is poorly-behaved, such that the MLM on non-FMP samples fails to bound the parameter on either side, just as the formal analysis suggested.

## Discussion

There is good news and bad news. The good news is that FIML MLM estimates of fixed level-1 coefficients are generally unbiased even in datasets that fail to meet the criteria of FMP sampling. Thus, one may use the MLM to control for macro-level nesting with such data.

However, the remaining news is very bad. First, many existing datasets fail to meet the criteria for FMP sampling for many geo-social contexts. Second, when this happens analysts estimate the wrong population parameter, which is biased as an estimator of the correct population parameter. Third, the bias can be substantial. Fourth, just as formal analysis indicated, the direction of bias is unknown such that estimates establish neither upper nor lower bounds. Fifth, the most damaged coefficients are often those that provide theoretical motivation for MLM estimation in the first place. Sixth, because non-FMP samples are non-probability samples, and because the wrong population parameter has been estimated, the standard errors are inappropriate, rendering hypothesis tests and out-of-sample inference indefensible. And, seventh, because fixed level-1 FIML coefficients are unbiased even as macro-level coefficients remain biased, obtaining expected level-1 findings cannot validate the macro-level portions of the model.

The simulations indicate that key coefficients are biased. The formal analysis anticipated this result, which is sensible because non-FMP samples are non-probability samples. For a single-level analysis there would be little dispute about the implications of using non-probability sampling–biased coefficients and inappropriate statistical inference; statistical analyses of such data would be justified only for exploratory, emergency, or hidden-population investigation, and even then publication would be rare. Yet, many multilevel analyses using non-FMP samples have been published, suggesting analysts believe these limitations are irrelevant once multiple levels of analysis are involved. Our result—that key multilevel estimates are indeed biased, and perhaps substantially so—confirms that non-FMP designs damage estimates, which should dissolve any confidence one might have in the model to succeed under such inhospitable conditions.

With respect to the standard error, I formally assessed whether non-FMP samples reference the correct population parameter—they do not—and formally and empirically investigated the existence of bias in the estimator. The results indicate that the standard error does not measure the precision of an estimate of the target population parameter. Thus, the findings indicate that hypothesis tests analysts have reported have little value.

One might take solace in that mean level-2 point estimates in the simulations are correctly signed. Such solace would be a mistake. The formal analysis indicated that the sign of the true MLM parameter is not generally recoverable from non-FMP sample results, because there are too many unknowns. Comparison with the case of selection bias is informative.

It is known that selection bias biases coefficients toward zero (Berk 1983). Thus, selection bias is well-behaved in that selection-biased estimates provide a floor for positive parameters and a ceiling for negative parameters. One might therefore reasonably regard selection-biased estimates as indicating the correct direction of the association.^19^

Such is not the case here. The bias is poorly-behaved—sometimes it pushes the coefficient closer to zero, sometimes it pushes the coefficient further from zero. Providing neither a floor nor a ceiling, MLM estimates based on non-FMP samples provide unreliable information on the sign of the coefficients of interest.

The findings indicate severe limits on use of multilevel modeling. As noted earlier, the MLM is not the first statistical model to require specific, more difficult-to-collect data. However, the MLM may be the first model used to contribute deeply to multiple substantive literatures prior to wide recognition of the heightened demands the model makes for sample design.

The findings reported here support yet again the classic truism: data collection is costly, challenging, and consuming; still, it is vastly easier to collect appropriate data than to specify a statistical model that attempts to salvage compromised data afterwards. In that spirit, the best counsel is to design and/or use FMP samples only, at least unless and until analysts devise ways of addressing the problem statistically. Although recognition of the problem of non-FMP samples may spur efforts to develop statistical models to “fix” the data, evidence suggests such efforts will be difficult (Stolzenberg and Relles 1990, 1997). Fortunately, here the data collection solution—collect FMP samples—can be implemented immediately, and requires no hard-to-justify model specifications or untestable statistical assumptions.

Stepping back, a broader view would observe that inadvertent use of non-probability samples is only one way in which MLMs go awry. Others have pointed to difficulties obtaining identification (i.e., the reflection problem) and their exacerbation when aggregated individual-level variables are used to measure macro-level factors (Hauser 1969, 1974; Manski 1995). While some continue to extend the MLM decade by decade—Goldstein et al. (1994) offer a MLM for time-series analysis; Steele et al. (2004) offer a competing risk event history analysis multilevel model—identification and measurement challenges remain largely unaddressed. Indeed, some analysts recommend the same procedure—aggregation of level-1 characteristics to measure level-2 characteristics (Hox 2010, p. 360)—that necessarily renders parameters unidentified (Manski 1995, pp. 129–133). Thus, as estimable MLMs proliferate, published empirical analyses may increasingly contain misleading findings owing to under-theorized multilevel measurement or unwitting failures of identification. To these two key challenges for the MLM we must add sample design as an issue multilevel analysts need explicitly address.

Despite the feasible solution of using FMP samples, the findings may still be quite deflating to some, especially as the findings arguably imply that many current “facts” in the field lack empirical foundation. At this point it is worth recalling Manski’s observation concerning attitudes toward methodological research. Manski noted that:

Empirical researchers usually enjoy learning of positive methodological findings. Particularly pleasing are results showing that conventional assumptions, when combined with available data, imply stronger conclusions than previously recognized. Negative findings are less welcome. Researchers are especially reluctant to learn that, given the available data, some conclusions of interest cannot be drawn unless strong assumptions are invoked. Be that as it may, both positive and negative findings are important to the advancement of science. (Manski 1995, p. 3).

Knowledge should not be deflating, for knowledge is power and thus is empowering. Once we recognize that many probability sample designs produce non-probability samples for the MLM, we need no longer waste time estimating MLMs on data for which findings will be biased in an unknown direction and for which any hypothesis tests are unjustified. Our recognition of the data demands of the model should reduce the role of biased findings in sculpting and unknowably warping our substantive understanding.

But these advantages only accrue if we accept that despite the wide availability of probability samples one cannot assume that probability samples are FMP samples for any given application of the MLM. Acceptance of this fact encourages analysts to ask: Is a specific dataset a fully multilevel probability sample for the geo-social level(s) of interest? If one seeks to draw inferences about multilevel parameters, then to proceed with the analysis, or accept the findings of already published analyses, the answer must be yes. By addressing this important question analysts may avoid relying upon what, in the end, and for our developing substantive and theoretical knowledge, turn out to be truly inconvenient datasets.

## Appendix

**Table 6:** Zero-order correlation matrices for the Monte Carlo simulation data

Panel A—study 1 population					
	Y1	Y2	X1	X2	Z1
Y1	1.000				
Y2	0.721	1.000			
X1	0.254	0.270	1.000		
X2	0.856	0.831	0.200	1.000	
Z1	0.013	0.013	0.003	0.001	1.000
Feeder to HS	0.033	0.036	0.136	0.027	0.012

Panel B—study 2 population						
	Y1	Y2	Minority	X1	X2	Z1
Y1	1.000					
Y2	.116	1.000				
Minority	$-$.237	$-$.150	1.000			
X1	.406	.117	$-$.446	1.000		
X2	.308	.324	$-$.444	.250	1.000	
Z1	.203	$-$.006	$-$.070	.038	.095	1.000

## Footnotes

1 Consistent with the term *level*, many analysts view the MLM as combining micro-level units nested in macro-level units. Yet, other data structures have been used (e.g., Bryk and Raudenbush 1988), such that this image of the MLM is too restrictive. Although I use the language of levels, the analysis applies broadly.

2 I ignore censuses because they do not pose these problems.

3 For example, multi-stage sampling of districts, schools, and then students means the inclusion probability of student *ij* is not independent of the inclusion probability of student $i+1, j$, which violates the independence criterion for SRS samples.

4 For example, U.S. researchers often use existing racial segregation to improve coverage of the national population. If simple random sampling were used the widely dispersed respondents would increase data collection costs and many samples might contain few members of some minority groups. To increase the number of minorities sampling statisticians often stratify geographic locations by racial/ethnic composition, thereby clustering respondents, reducing data collection cost, and facilitating statistically and logistically efficient cross-group comparisons (i.e., smaller standard errors and lower financial cost). However, some analysts have argued this approach mis-measures diversity within the minority group (Engram 1982).

5 One may set a given $\delta _\mathrm{{jk}} =$ 0 and include Z$_\mathrm{{j}}$ to estimate deterministically-varying $\beta _\mathrm{{jk}}$’s. Sample issues do not substantively change with this specification, so I do not discuss this further.

6 Indeed, if analysts use some function of Z$_\mathrm{ij,r}$ to measure Z$_\mathrm{j}$, then the independent variable Z will also be compromised, at least as a measure of a characteristic of unit $j$.

7 I thank an anonymous reviewer for this observation.

8 In Eq. 7 p$_\mathrm{{j}}$ reflects the proportion of persons in context $j$ who can be reached; exchangeability concerns n$_{j}$/N, the proportion of all sampled persons who are in context $j$.

9 NELS freshened the 1990 and 1992 samples to make them representative of sophomores/seniors in the nation at-large, not to make the in-school samples representative of each high school (Ingels et al. 1994).

10 In terms of the formal explication, taking the students in high school $$j$$, those from sampled eighth grade schools are in group $$r$$, and those from non-sampled eighth grade schools are in group $$u$$. The proportion of students in each group ($$p$$ and (1 – $$p))$$ varies across high schools.

11 In terms of the formal explication, persons in state $j$, for example, who are in the PSU for the region are in group $r$, whereas other persons in the state are in group $u$.

12 I agree the perfect should not be the enemy of the good. However, studies using samples biased in an unknown direction to an unknown and arguably unknowable degree are not “good.” Still, some may see a call to strike findings from the record as extreme. I appreciate the sentiment, but what other response is justifiable? One could argue such findings may be used to form bayesian priors or to suggest theory. With respect to bayesian analyses, bayesians may draw priors from anywhere, so such use is defensible. Yet, with “development of priors” as a standard, what analysis *cannot* be justified? We use probability samples because we know neither the population parameters nor all the unmeasured connections between measured and unmeasured factors. Holding non-probability sample results in hand—results with biases of unknown size and direction—how can one trust the findings even as a point of departure for other work or theory-development? Why should theory or “establishing a point of departure” *lower* our standards, given that both theory and our point of departure may greatly affect future findings? Indeed, might biased studies point analysts in the wrong direction, or raise ultimately less grounded questions, wasting the field’s time and journal space? Probability sampling partly preempts such problems. Thus, I contend, the better approach is to set aside such work currently in the literature and prevent publication of such work.

13 For Fuller (2008) year-specific wages are the micro-level units and persons are macro-level units. Geocode data measures, for example, the labor market conditions in which persons earned their wages. Inference is still to a unit/level for which probability sampling was used.

14 The X$_{1}$Z$_{1}$ and X$_{2}$Z$_{1}$ correlations are near zero because the MLM purports to partition $\sigma ^{2}_{Y}$ across levels. Setting correlations near zero increases the chance the model will work as claimed.

15 The social basis of these decisions is that attrition probability might be partly determined by whether the student and school match. Otherwise, attrition is random with respect to the other variables. In the real world high and low achievers, the wealthy and the poor fail to enter the fed high school. Hence, I did not specify a connection between attrition probability and the variables that stand-in for socioeconomic background and prior measured achievement.

16 I do not estimate a coefficient for the main effect of Z$_{1}$. In the MLM a main effect for Z$_{1}$ would be in the macro-level equation for $\beta _{0j}$. Equation 20 indicates that Y$_{2}$ was constructed with a main effect of zero for Z$_{1}$; thus, Z$_{1}$ should be omitted from the equation for $\beta _{0j}$.

17 I did not estimate QWMLM models in study 2.

18 As in study 1, I do not estimate a coefficient for the main effect of Z$_{1}$. In the MLM a main effect for Z$_{1}$ would be in the macro-level equation for $\beta _{0j}$. Equation 22 indicates that Y$_{2}$ was constructed with a main effect of zero for Z$_{1}$; thus, Z$_{1}$ should be omitted from the equation for $\beta _{0j}$.

19 To draw this conclusion one would have to hope one is not in the perverse situation of such strong selection bias as to push the estimate past zero, thus making the estimate wrongly signed as well. But, such perversities are likely rare, making selection-biased estimates of value.
